# Effects of Physical Exercises on Pulmonary Rehabilitation, Exercise Capacity, and Quality of Life in Children with Asthma: A Meta-Analysis

**DOI:** 10.1155/2021/5104102

**Published:** 2021-12-23

**Authors:** YiRan Liu, Yan Zhao, Fang Liu, Lin Liu

**Affiliations:** ^1^School of Sport and Health, Nanjing Sport Institute, Nanjing 210014, Jiangsu, China; ^2^Respiratory Medicine of Xuyi People's Hospital, Huaian 223001, Jiangsu, China

## Abstract

**Objective:**

This study aimed to systematically evaluate the effect of exercise on pulmonary function, exercise capacity, and quality of life in children with bronchial asthma.

**Methods:**

A comprehensive search was performed using PubMed, Cochrane Library, Web of Science, EBSCO, CNKI, and Wanfang Data Knowledge Service platform to identify any relevant randomized controlled trials (RCTs) published from inception to April 2021. The Cochrane risk of the bias tool was utilized to evaluate the methodological quality of the included studies, and RevMan 5.3 was applied to perform data analyses.

**Results:**

A total of 22 RCTs involving 1346 patients were included. The results of the meta-analysis showed that exercise had significant advantages in improving lung function and exercising capacity and quality of life in children with asthma compared with conventional treatment, such as the forced vital capacity to predicted value ratio (SMD = 0.27; 95% CI: 0.13, 0.40, and *P* < 0.0001), the peak expiratory flow to predicted value ratio (MD = 4.53; 95% CI: 1.27, 7.80, and *P*=0.007), the 6-minute walk test (MD = 110.65; 95% CI: 31.95, 189.34, and *P*=0.006), rating of perceived effort (MD = −2.28; 95% CI: −3.21, −1.36, and *P* < 0.0001), and peak power (MD = 0.94; 95% CI: 0.37, 1.52, and *P*=0.001) on exercise capacity and pediatric asthma quality of life questionnaire (MD = 1.28; 95% CI: 0.60, 1.95, and *P*=0.0002) on quality of life. However, no significant difference was observed in the forced expiratory flow between 25% and 75% of vital capacity (*P*=0.25) and the forced expiratory volume at 1 second to predicted value ratio(*P*=0.07).

**Conclusions:**

Current evidence shows that exercise has a certain effect on improving pulmonary function recovery, exercise capacity, and quality of life in children with bronchial asthma. Given the limitation of the number and quality of included studies, further research and verification are needed to guide clinical application.

## 1. Introduction

Bronchial asthma is a common chronic respiratory disease in children, and the etiology is unclear. Most studies indicate that asthma is related to genetic and environmental factors, such as allergic constitution, viral infection, particulate matter, smoke, and ozone exposure [1]. The main clinical manifestations of asthma include breathlessness, cough, shortness of breath, and wheezing, which are easy to attack or aggravate at night and in the early morning [2] and need long-term treatment because of repeated attacks and prolonged treatment, which may lead to irreversible damage to lung function. In severe cases, these manifestations will affect the growth, development, exercise ability, and quality of life of children. The number of patients with global bronchial asthma increases annually with the rapid pace of modern society and the progress of science and technology, particularly in children [3]. According to the statistics, at present, more than 300 million children suffer from bronchial asthma worldwide, and global analysis of the ISAAC study and other reports showed that the prevalence of asthma averaged approximately 10% for 6–7 years old [4]. Therefore, effectively preventing and treating childhood asthma has become a focus of global research.

At present, the prevention and treatment of asthma are primarily based on drugs, such as glucocorticoids, antihistamines, *β*-2 agonists, and leukotriene receptor antagonists [5]. However, the effect of most drugs is limited, and they will have several side effects; therefore, finding a safe and effective alternative therapy has become the focus of pulmonary physicians. In recent years, considerable attention has been paid to the role of exercise in alleviating airway inflammation and delaying or reversing airway remodeling. During the outbreak of novel coronavirus pneumonia (COVID-19) in China, exercise, as an important means for the recovery of pulmonary function of COVID-19 patients, is highly recommended by medical staff during the development of the epidemic. It is also included in COVID-19's rehabilitation guidelines for integrated traditional Chinese and Western medicine (first edition) [6]. Based on the Global Initiative for Asthma (GINA) guidelines, children with asthma should participate in physical exercises like normal children, which is considered to be an important part of the nondrug prevention and treatment strategy for children with asthma. Therefore, exercise has also been used in the rehabilitation of lung function, exercise ability, and quality of life in children with bronchial asthma, but the prevention and treatment effects are different [7–12]. Andrade et al. [11] conducted a 6-week randomized controlled trial (RCT) on 33 children with moderate asthma. The study showed that aerobic exercise intervention had significant advantages over nonexercise in improving asthma-related symptoms, lung function, exercise ability, and quality of life. However, exercise was once regarded as the inducing factor of asthma, particularly in patients with exercise-induced asthma, and children would reduce or give up exercise because of repeated attacks. Meta-analysis shows that swimming for 6–12 weeks is significantly better than routine treatment in improving pulmonary function indexes such as FEV1% pred and FEF25–75% pred in children with bronchial asthma, but no significant difference was observed in FVC% pred, FEF50% pred, and other indexes [12]. A previous systematic review on 11 studies by Zhang et al. [13] found physical training improved FVC (% pred) significantly in children with asthma. This finding may support the therapy of physical training in asthmatic children. But further research involving the physical training mode, the duration, frequency, and other outcome indicators is needed. Therefore, the extensive application of exercise in children with bronchial asthma is difficult.

To date, whether exercise can be included in the rehabilitation of lung function in children with bronchial asthma and whether exercise can improve children's pulmonary function, exercise ability, and quality of life remains unknown. Therefore, this paper intends to systematically evaluate the intervention effect of exercise on the improvement of lung function, exercise ability, and quality of life in children with bronchial asthma by meta-analysis, thereby objectively and quantitatively evaluating the effectiveness of exercise rehabilitation and providing a scientific and rigorous basis for clinical respiratory and rehabilitation doctors in their clinical work and scientific research.

## 2. Materials and Methods

This systematic review was conducted and reported in accordance with the Preferred Reporting Items for a Systematic Review and Meta-Analysis Framework [14, 15]. We did not publish or register a protocol for this study.

### 2.1. Search Strategy

A predetermined search strategy was implemented until April 2021 using the PubMed, Cochrane Library, Web of Science, EBSCO, China Knowledge Network, and Wanfang data knowledge service platform for RCTs on the effect of exercise on children with bronchial asthma. Search strategies included a combination of text words and subject headings (such as MeSH and free terms) relating to (1) physical activity or exercise; (2) asthma; (3) child; and (4) random or allocation. We did not explicitly include search terms related to pulmonary rehabilitation or exercise capacity in the search strategies as these terms were rarely mentioned in the title, abstract, or subject headings, but they were often embedded within full-text articles; thus, they must be identified at the screening level.

### 2.2. Study Selection

We considered the participant, intervention, comparison, outcome, time, and study (PICOTS) design criteria to ascertain the study inclusion criteria. The inclusion criteria comprised the following: (1) Types of studies: This meta-analysis included only RCTs about the effects of exercise and routine exercise prescriptions on children and adolescents with bronchial asthma. (2) Research subjects: All met the revised standard of the Respiratory Group of the Chinese Medical Pediatrics Association [16] or the diagnostic standard of GINA children with asthma [17]. In addition, the disease was in the nonacute stage, and the sex, race, and nationality of the subjects were not restricted. (3) Intervention: Exercise was based on routine treatment, including aerobic training (swimming, ball games, rope skipping, jogging, and cycling), strength training, and balance and coordination training. (4) Control: Routine treatment includes medication, nutritional support, family breathing exercises, or routine activities without exercise. (5) Outcome: Pulmonary function indices include the forced vital capacity to predicted value ratio (FVC% pred), the forced expiratory volume at 1 s to predicted value ratio (FEV1%pred), the forced expiratory flow between 25% and 75% of vital capacity ratio (FEF25–75% pred), and the peak expiratory flow to predicted value ratio (PEF%pred). Exercise capacity indices include the 6-minute walk test (6MWT), rating of perceived effort (RPE), and peak power (PP). Quality of life indicators include Pediatric Asthma Quality of Life Questionnaire (PAQLQ): symptom score, activity score, and emotional score.

The exclusion criteria comprised the following: (1) reviews, guidelines, letters, commentaries, book chapters, or published only as an abstract or poster; (2) studies of incomplete design; (3) full-text literature that is not available through various channels and methods; (4) research articles of poor quality and lack of access to quality information; and (5) the sample size of less than 10 cases.

### 2.3. Data Extraction

The two researchers independently screened the literature on the basis of the admission and exclusion criteria, extracted the data, checked each other after the completion of the statistics, and discussed the main points or referred to the opinions of the third party in case of disagreement. Extraction data include first author, year of publication, research methods, sample size, intervention measures, treatment frequency, treatment time, and outcome index. For trials with more than two intervention groups, the experimental group was compared with the control group by combining the data of all relevant control groups [18]. If required information was not reported, then we tried to request it from the corresponding author of the studies and exclude the literature if no other channel information was available or the author had no response [18].

### 2.4. Quality Assessment

Two reviewers were involved in the risk of bias assessment. The risk of bias was assessed using the Cochrane Collaboration tool, which contained six domains (random sequence generation, allocation concealment, blinding of participants and personnel, blinding of outcome assessors, incomplete outcome data, selective reporting, and other sources of bias) [18]. A judgement was made regarding the risk of bias for each of these domains using three categories “low,” “high,” and “unclear” if the study details were insufficient. Any disparity regarding risk of bias was discussed and resolved by consultation with the principal author.

### 2.5. Statistical Analysis

We performed a preliminary narrative synthesis of the data. Depending on the heterogeneity of the studies, we performed either a fixed-effect or a random effect meta-analysis. The heterogeneity of the trials was assessed through visual inspection of forest plots and the calculation of the *I*^2^ statistic using a 50% limit to indicate substantial heterogeneity [19]. End point scores were expressed as standardized mean differences (SMDs) with associated 95% confidence intervals (CIs). We performed the meta-analysis using RevMan 5 software [20]. The risk of publication bias was assessed graphically by a funnel plot [21], and the robustness of the overall estimates obtained was assessed by sensitivity analyses.

## 3. Results

### 3.1. Characteristics of Included Studies

**Figure 1:**
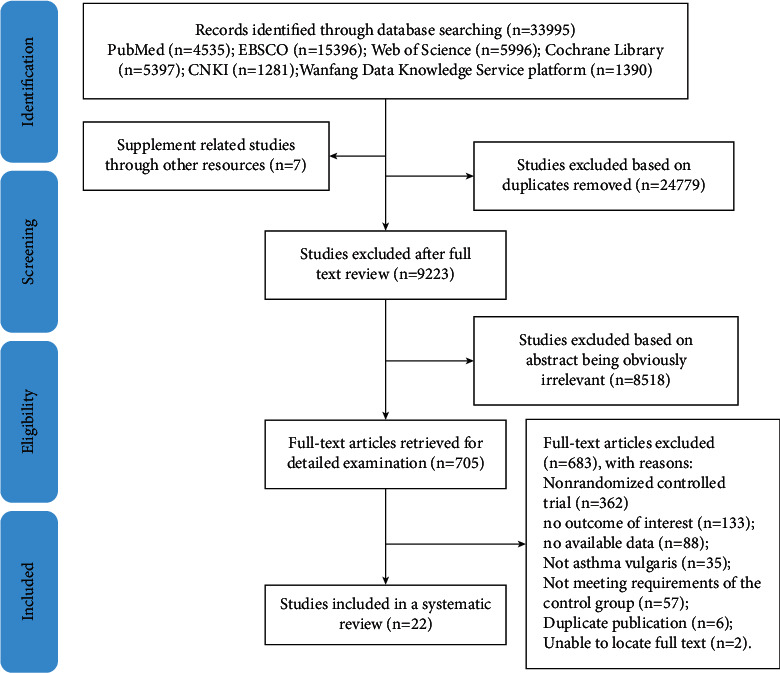
Literature screening process and results.

A total of 33,995 references were initially identified from the computerized search. After removing 24,779 duplicates, the titles of the remaining 9223 articles were screened, and 1004 abstracts were retrieved for further scrutiny. A total of 705 studies were selected and retrieved for a full review, of which 688 were excluded. Finally, 22 studies met the criteria as described in the “Methods,” which were included in the analysis [11, 22–42]. The flow chart of literature retrieval and screening is shown in Figure 1.  Table 1 summarizes the main characteristics of the included studies. A total of 1346 study subjects were included in the meta-analysis.

### 3.2. Risk of Bias in Included Studies

See Figures S1 and S2 in the Supplementary Material for the risk-of-bias assessment for included studies. Twenty out of 22 studies (90%) were RCTs, with a low risk of bias for allocation. However, 17 of these RCTs did not provide details about the allocation concealment; thus, this item was considered with an unclear risk of bias. Given the nature of intervention in these studies (physical exercise), blinding of participants was not possible. In addition, blinding of outcome assessment was performed in two studies, whereas the remaining 20 studies had an unclear risk of bias for this item. Therefore, the overall quality was medium.

### 3.3. Meta-Analysis Results

#### 3.3.1. Studies of the Effect of Physical Exercises on Pulmonary Rehabilitation in Children with Asthma

**Figure 2:**
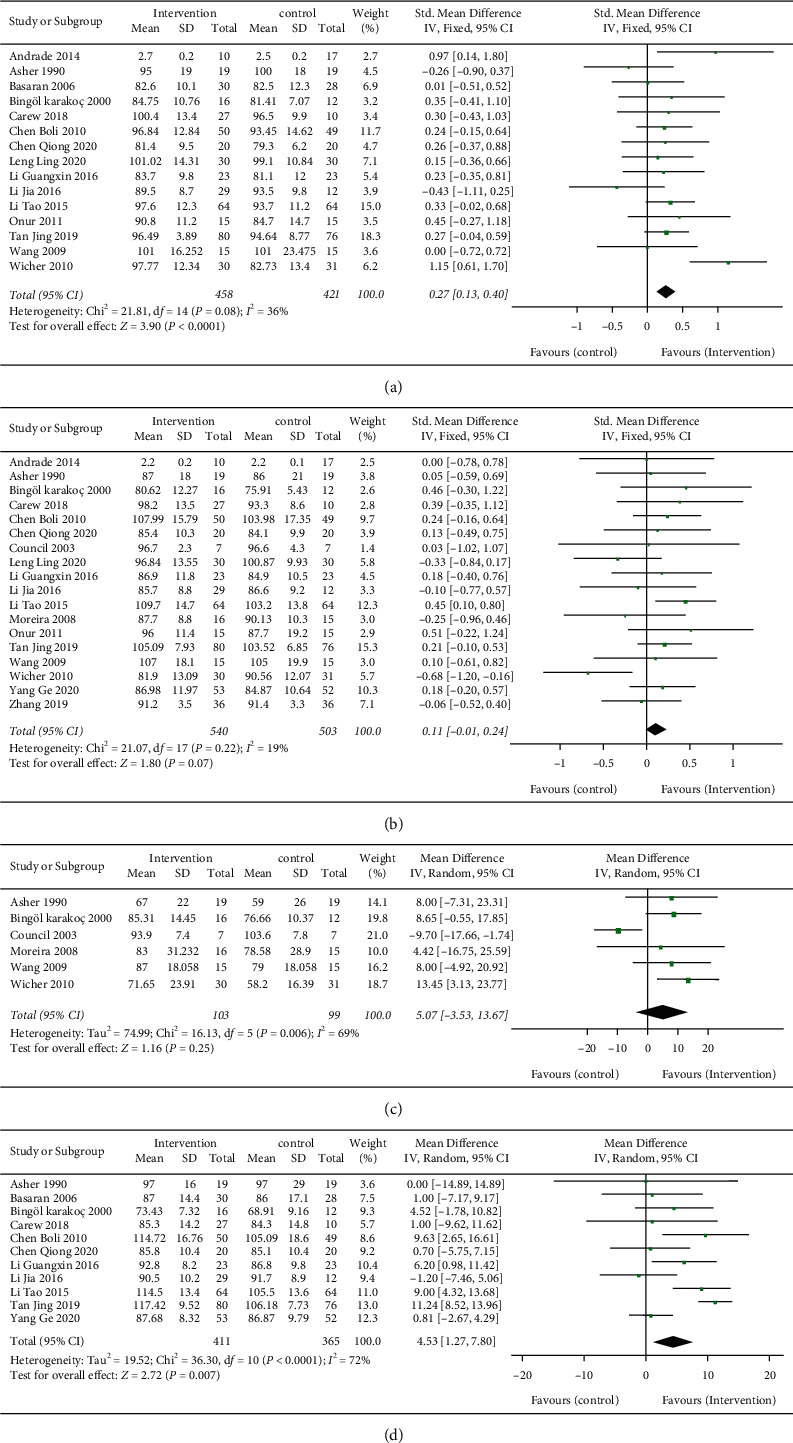
The effect of exercise on pulmonary rehabilitation. The effect of exercise on (a) FVC%pred index; (b) FEV1%pred index; (c) FEV25–75%pred index; and (d) PEF%pred index.

Fifteen studies evaluated the effects of physical exercise on FVC% pred in children with asthma. A pooled analysis of the heterogeneous data (*I*^2^ = 36%; *P* < 0.0001) included a total of 458 intervention and 421 control participants. As shown in Figure 2(a), exercise has a significant advantage in improving FVC% pred in children with bronchial asthma compared with the control group, and the difference among the groups is statistically significant (SMD = 0.27 and 95% CI: 0.13 to 0.40). Eighteen studies evaluated the effects of physical exercise on FEV1% pred in children with asthma. A pooled analysis of the heterogeneous data (*I*^2^ = 19%; *P* > 0.05) included a total of 540 intervention and 503 control participants. The results of Figure 2(b) showed that the significant effect of exercise on improving FEV1% pred indexes in children with bronchial asthma cannot be determined compared with the control group, and no significant difference was observed between the two groups (SMD = 0.11 and 95% CI: −0.01 to 0.24). Six studies evaluated the effects of physical exercise on FEV25–75% pred in children with asthma. A pooled analysis of the heterogeneous data (*I*^2^ = 69%; *P* > 0.05) included a total of 103 intervention and 99 control participants. As shown in Figure 2(c), the significant effect of exercise on improving FEV25–75% pred indexes in children with bronchial asthma cannot be determined compared with the control group, and no significant difference was observed between the two groups (MD = 5.07 and 95% CI: −3.53 to 13.67). Eleven studies evaluated the effects of physical exercise on PEF% pred in children with asthma. A pooled analysis of the heterogeneous data (*I*^2^ = 72%; *P* < 0.01) included a total of 411 intervention and 365 control participants. As shown in Figure 2(d), compared with the control group, exercise had significant advantages in improving PEF% pred indexes in children with bronchial asthma, and the difference among groups was statistically significant (MD = 4.53 and 95% CI: 1.27 to 7.80).

#### 3.3.2. Studies of the Effect of Physical Exercises on Exercise Capacity in Children with Asthma

Five studies evaluated the effects of physical exercise on 6 MWT in children with asthma. A pooled analysis of the heterogeneous data (*I*^2^ = 96%; *P* < 0.01) included a total of 132 intervention and 124 control participants. As shown in Supplementary Figure S3, exercise has significant advantages in improving the exercise ability of children with bronchial asthma compared with the control group, and the difference among the groups is statistically significant (MD = 110.65 and 95% CI: 31.95 to 189.34). The heterogeneity for 6MWT was reduced from 96% to 38% after the exclusion of the study by Latorre-Roma'n et al. [29].

Only three studies evaluated the effects of physical exercise on RPE in children with asthma. A pooled analysis of the heterogeneous data (*I*^2^ = 61%; *P* < 0.001) included a total of 92 intervention and 79 control participants. As shown in Supplementary Figure S4, exercise has significant advantages in improving the exercise ability of children with bronchial asthma compared with the control group, and the difference among the groups is statistically significant (MD = −2.28 and 95% CI: −3.21 to −1.36). The heterogeneity for RPE was reduced from 61% to 0% after the exclusion of study by Latorre-Roma'n et al. [29].

Only three studies evaluated the effects of physical exercise on PP in children with asthma. A pooled analysis of the heterogeneous data (*I*^2^ = 69%; *P*=0.001) included a total of 83 intervention and 82 control participants. As shown in Supplementary Figure S5, exercise has significant advantages in improving the exercise ability of children with bronchial asthma compared with the control group, and the difference among the groups is statistically significant (MD = 0.94 and 95% CI: 0.37 to 1.52). The heterogeneity for PP was reduced from 69% to 0% after the exclusion of the study by Li et al. (2016) [31].

#### 3.3.3. Studies of the Effect of Physical Exercises on Quality of Life in Children with Asthma

**Figure 3:**
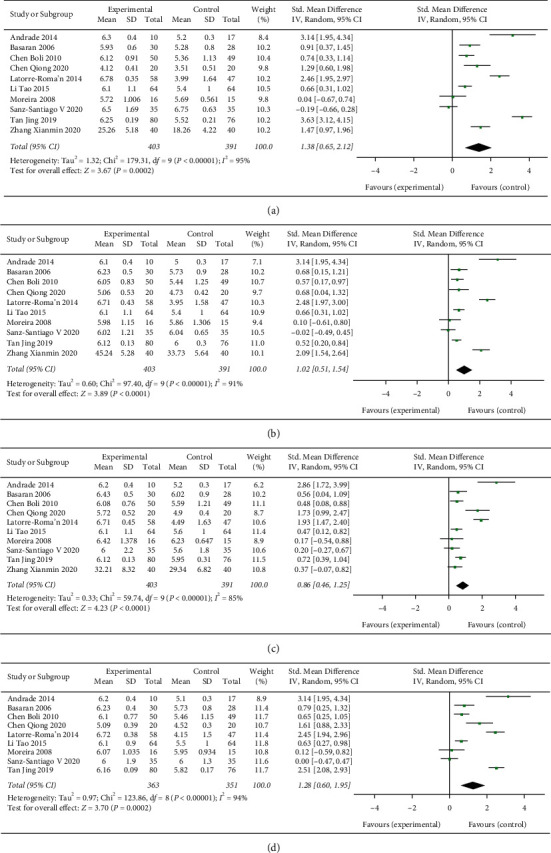
The effect of exercise on the quality of life. The effect of exercise on (a) activity score; (b) symptom score; (c) emotional score; and (d) total score.

Ten studies evaluated the effects of physical exercise on quality of life in children with asthma. A pooled analysis of the heterogeneous data (*I*^2^ = 94%; *P* < 0.001) included a total of 363 intervention and 351 control participants. As shown in Figure 3, exercise has significant advantages in improving the quality of life of children with bronchial asthma compared with the control group, and the difference between the groups is statistically significant (MD = 1.28 and 95% CI: 0.60 to 1.95).

### 3.4. Subgroup Analysis

According to the subgroup analysis of different intervention times, the results showed that the exercise intervention of more than 8 weeks was significantly better than the routine control group in improving the indexes of FVC% pred, FEV1% pred, FEV25–75% pred, and PEF% pred in children with bronchial asthma, and the difference was statistically significant (Table 2).

### 3.5. Sensitivity Analysis

**Figure 4:**
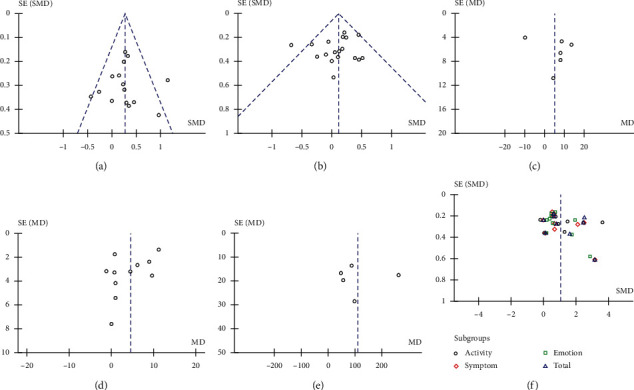
Funnel plot of the meta-analysis of the data. The funnel plot of (a) FVC% pred; (b) FEV1% pred; (c) FEV25–75% pred; (d) PEF% pred; (e) 6MWT; and (f) Quality of life scoring index.

According to the strict quality evaluation, the 22 articles included were selected to draw the funnel chart with the number of observation indexes ≥5, such as FVC% pred, FEV1% pred, FEV25–75% pred, PEF% pred, 6MWT, and quality of life score (Figure 4). The asymmetry of the funnel chart can be seen from the chart, which may be related to the inconsistency of specific intervention methods, treatment frequency, and outcome measurement methods in the included RCTs; that is, real heterogeneity might be observed among studies, not necessarily publication bias.

## 4. Discussion

In recent years, the prevalence rate of bronchial asthma in children around the world has increased significantly, and the overall control level remains unsatisfactory [43]. If we cannot get timely and effective treatment, then the condition of some patients will continue into adulthood, which brings a huge economic burden to the family and a huge consumption of medical and health resources to the country [16]. Therefore, the prevention and treatment of bronchial asthma in children should be performed as early as possible and should adhere to the principles of long-term persistence, standardization, and personalized treatment [44]. Exercise improves healthy growth and development of children, but exercise in children with asthma remains a great concern because they have asthma-related symptoms in the course of exercise and they may avoid exercise because of fear. In addition, given the vague understanding of children with asthma participating in exercise, parents will not encourage or even restrict their children to participate in exercise. Therefore, whether exercise can be used as a complementary alternative therapy for the rehabilitation of lung function in children with bronchial asthma and whether it can effectively improve the exercise ability and quality of life of children lacks relevant reports at home and abroad [12, 22, 45]. Further promoting the orderly application of exercise intervention in the field of rehabilitation of chronic respiratory diseases promotes not only the universal development of sports in the whole process of prevention, treatment, and rehabilitation of respiratory diseases, but also the high-quality development of medical and physical integration. In this study, through evidence-based medicine, the quantitative results show that exercise is significantly better than routine treatment in improving exercise ability, lung function, and quality of life of children with bronchial asthma, and the difference is statistically significant.

With the deepening of the study of exercise in children with bronchial asthma, the relevant research conclusions are still controversial. The results of this study show that the pulmonary function index, as an important adjustment index for the evaluation, treatment, and severity monitoring of bronchial asthma, has always been the focus of respiratory doctors [46]. In this study, we collected 22 items of RCT and evaluated the effectiveness of exercise and routine treatment in interfering with the recovery of pulmonary function in children with bronchial asthma. The results showed that exercise was significantly better than routine treatment in improving FVC% pred, PEF% pred, and other pulmonary function indexes in children with asthma(*p* < 0.01). However, no significant difference was observed in the improvement of FEV1% pred and FEF25–75% pred between the two groups(*p* > 0.05). Subgroup analysis further showed that the exercise intervention of more than 8 weeks was significantly better than routine treatment in improving the indexes of FVC% pred, FEV1% pred, FEV25–75% pred, and PEF% pred in children with bronchial asthma. Therefore, the exercise intervention time for children with asthma should be maintained at more than 8 weeks.

The decrease of physical fitness (such as muscle strength and endurance and cardiopulmonary function) caused by the decrease of physical activity in children with bronchial asthma is an important factor in the decline of their quality of life and control of the disease [47]. Therefore, improving exercise ability and improving quality of life play a positive role in alleviating asthma symptoms. The 6-minute walking test (6MWT) is a simple, safe, and well-tolerated submaximal exercise test for adults and children with chronic heart or respiratory diseases. It is an effective tool to reflect the exercise ability and disease severity of subjects by measuring their walking distance within 6 min [48]. In this study, compared with the control group, the 6MWT distance of the exercise group increased, and a significant difference was observed(*p* < 0.001). The fatigue degree (RPE) of the exercise group after exercise was lower than that of the control group(*p* < 0.001). Therefore, exercise can improve the exercise ability of children with asthma and increase their exercise endurance in daily life. In addition, the 30 s Wingate test with an anaerobic power bicycle was used to detect the exercise ability of children with asthma, and the obtained PP index could reflect the children's anaerobic exercise ability. In this study, the PP of the exercise group was significantly different from that of the control group(*p*=0.001), suggesting that scientific and effective exercise training can improve the cardiopulmonary system oxygen transport capacity of children with asthma and benign adaptation of the body. Consequently, the exercise ability has been improved [49]. Philipp et al. [50] have also shown that exercise training can effectively relieve asthma symptoms and improve exercise ability.

The Pediatric Asthma Quality of Life Questionnaire is a scale to evaluate the quality of life of children with asthma from 7 to 17 years old. It has high credibility, and it can accurately reflect the changes of the disease [51]. In this study, the observed symptoms, limitations of activity, emotional function, and total score of the exercise group were significantly different from those of the control group(*p* < 0.001), and the score was significantly higher than that of the control group, indicating that scientific and reasonable exercise training can improve the quality of life of children with asthma. This result is similar to that of the study by Fanelli et al. [52] on 38 children with moderate and severe persistent asthma for 16 weeks and Basaran et al. [23] on 62 children with mild-to-moderate asthma for 8 weeks. The results are the same as the results of the studies of aerobic training for 16 weeks in 38 children with moderate and severe persistent asthma and 8 weeks of moderate basketball training in 62 children with mild and moderate asthma.

The inclusion of literature in this meta-analysis still has limitations. First, a small number of included articles and research objects are identified, and some problems are found in the random grouping and blind implementation of some RCTs, which makes the research easy to be disturbed by subjective factors and affects the authenticity and reliability of the results. Second, in the intervention programs included in the study, the duration and frequency are not consistent, and the course of disease at the beginning of the intervention is also different, which has become an important factor affecting the curative effect to a certain extent. In addition, differences are observed in the rating scales and measurement methods used to measure outcome indicators, which leads to a decrease in the credibility of the results of the meta-analysis.

## 5. Conclusion

This meta-analysis of 22 randomized controlled trials shows that reasonable and effective exercise can significantly improve lung function, exercise ability, and quality of life in children with bronchial asthma. It also confirmed the effectiveness of exercise in these pulmonary function, exercise ability, and quality of life scores, which can be used as a reference basis for clinical exercise rehabilitation of children with asthma. However, considering the great differences in the specific treatment mode, the time, frequency, and course of disease at the beginning of intervention among different studies are limited by the quality and quantity of the included studies, and the specific clinical effects are still controversial. Therefore, conducting high-quality, large sample size RCTs is necessary for verification and evaluation in the future.

## Figures and Tables

**Table 1:** Characteristics of studies included.

First author. (year).(reference)	Sample size (I/C)	Mean age (^*∗*^I/C)	Intervention	Control	Intervention duration	Intervention frequency	Outcome measure
Andrade et al. (2014). [11]	27 (10/17)	11.4 ± 2.3/ 11.7 ± 2.3	Aerobic training	Routine activities	6 w	3 times/week, 20–30 minutes/time	FVC% pred, FEV1% pred, PAQLQ, 6MWT
Asher et al. (1990). [22]	38 (19/19)	9.5 ± 2.7/ 10 ± 2.6	Physical therapy based on exercise	Routine drug therapy	2 w	2 times/day, 20–30 minutes/time	FVC% pred, FEV1% pred, FEF25–75% pred, PEF% pred
Basaran et al. (2006). [23]	58 (30/28)	10.35 ± 2.2/ 10.45 ± 2.1	Submaximal aerobic exercise	Routine activities	8 w	3 times/week, 55–60 minutes/time	FVC% pred, PEF% pred, PAQLQ, 6MWT
Karakoç et al. (2000). [24]	28 (16/12)	10.8 ± 2.3/ 10.2 ± 2.4	Lung rehabilitation training program based on exercise	Routine activities	4 w	—	FVC% pred, FEV1% pred, FEF25–75% pred, PEF% pred
Carew et al. (2018). [25]	37 (27/10)	13.3 ± 1.9, 13.2 ± 1.6, 13.5 ± 1.8/ 12.0 ± 3.1	Swimming, football, basketball	Routine activities	6 w	Once a week, 40 minutes	FVC% pred, FEV1% pred, PEF% pred
Chen. (2010). [26]	99 (50/49)	9.10 ± 2.14/ 9.18 ± 3.27	Exercise prescription	Routine drug therapy	16 w	3 times/week, 20 minutes/week	FVC% pred, FEV1% pred, PEF% pred, PAQLQ
Chen et al. (2020). [27]	40 (20/20)	11.4 ± 2.8/ 12.1 ± 3.4	Fixed treadmill exercise	Routine activities	12 w	3 times/week, 30 minutes/week	FVC% pred, FEV1% pred, PEF% pred, 6MWT, RPE, PAQLQ
Counil et al. (2003). [28]	14 (7/7)	14 ± 0.6/ 13.9 ± 0.8	Power cycling	Routine activities	6 w	3 times/week, 45 minutes/week	FEV1% pred, FEF25–75% pred, PP
Latorre-Roma'n et al. (2014). [29]	105 (58/47)	11.55 ± 1.01/ 11.51 ± 1.42	Indoor interval training	Routine activities	12 w	3 times/week, 60 minutes/week	PAQLQ, 6MWT, RPE
Leng et al. (2020). [30]	60 (30/30)	6.72 ± 2.55/ 6.59 ± 2.18	Exercise prescription	Routine drug treatment and massage	12 w	5 times/week, 60 minutes/week	FVC% pred, FEV1% pred
Li et al. (2016). [31]	46 (23/23)	11.9 ± 2.3/ 12.5 ± 3.0	Intermittent anaerobic exercise (pedaling)	Routine activities	8 w	3 times/week, 30 seconds/time	FVC% pred, FEV1% pred, PEF% pred, PP
Li et al. (2016). [32]	41 (29/12)	11.7 ± 2.0, 12.5 ± 1.6/ 10.8 ± 1.3	Medium intensity continuous exercise; high intensity intermittent exercise	Routine activities	8 w	4 times/week, 40 minutes/time or 30 seconds/time	FVC% pred, FEV1% pred, PEF% pred, 6MWT, RPE
Li et al. (2015). [33]	128 (64/64)	8.8 ± 2.4	Exercise prescription	Routine drug therapy	24 w	3 times/week, 20 minutes/week	FVC% pred, FEV1% pred, PEF% pred, PAQLQ
Moreira et al. (2008). [34]	31 (16/15)	12.9 ± 3.4/ 12.5 ± 3.5	Submaximal exercise (aerobic exercise, strength training, balance and coordination training, relaxation exercise)	Routine activities	12 w	2 times/week, 50 minutes/time	FEV1% pred, FEF25–75% pred, PAQLQ
Onur et al. (2011). [35]	30 (15/15)	9.8 ± 1.8/ 10.3 ± 2.0	Cycling	Routine drug therapy	8 w	2 times/week, 60 minutes/time	FVC% pred, FEV1% pred
Sanz-Santiago et al. (2020). [36]	60 (35/35)	12.1 ± 2.1/ 11.1 ± 2.9	Resistance and aerobic training	Routine activities	12 w	3 times/week, 20–40 times/week,	PAQLQ
Tan. (2019). [37]	156 (80/76)	8.24 ± 2.13/ 8.08 ± 2.17	Swimming, intermittent sports (table tennis, badminton, basketball, etc.)	Routine drug therapy	12 w	3 times/week, 20–30 minutes/time	FVC% pred, FEV1% pred, PEF% pred, PAQLQ
Wang et al. (2009). [38]	30 (15/15)	7–12	Swimming	Routine activities	6 w	3 times/week, 50 minutes/week	FVC% pred, FEV1% pred, FEF25–75% pred
Wicher et al. (2010). [39]	61 (30/31)	10.35 ± 3.13/ 10.90 ± 2.63	Swimming	Routine drug therapy	12 w	2 times/week, 60 minutes/time	FVC% pred, FEV1% pred, FEF25–75% pred
Yang et al. (2020). [40]	105 (53/52)	8.24 ± 1.27/ 8.68 ± 1.36	Intermittent anaerobic exercise training	Routine breathing training	8 w	7 times/week, 30 minutes/time	FEV1% pred, PEF% pred, pp
Zhang et al. (2019). [41]	72 (36/36)	6.9 ± 2.3/ 7.1 ± 2.7	Aerobic training	Routine drug therapy	6 w	3 times/week, 40 minutes/week	FEV1% pred
Zhang et al. (2020). [42]	80 (40/40)	8.2 ± 2.5/ 7.9 ± 2.2	Aerobic exercise and anaerobic exercise	Routine activities and health education	8 w	3–5 times/week, 35–50 minutes/week	PAQLQ

I: intervention group; C: control group; w: week; FVC% pred: pulmonary function indices include forced vital capacity to predicted value ratio; FEV1% pred: forced expiratory volume at 1 s to predicted value ratio; FEF25–75% pred: forced expiratory flow between 25% and 75% of vital capacity ratio; PEF% pred: peak expiratory flow to predicted value ratio; PAQLQ: Pediatric Asthma Quality of Life Questionnaire; 6 MWT: the 6-minute walk test; RPE: rating of perceived effort; PP: peak power; —: no data.

**Table 2:** Subgroup analysis results.

Subgroup (intervention time)	Studies	Heterogeneity test results		Effect model	Meta-analysis results	
		*P*	*I* ^2^ (%)		MD/SMD (95% CI)	*P*value
FVC% pred						
≥8 w	10	0.01	56	Fixed	2.62(1.19, 4.04)	0.0003
<8 w	5	0.21	32	Fixed	0.20(−0.12, 0.53)	0.22

FEV1% pred						
≥8 w	11	0.02	52	Fixed	1.09(−0.38, 2.56)	0.15
<8 w	7	0.92	0	Fixed	0.10(−0.15, 0.36)	0.42

FEV25–75% pred						
≥8 w	2	0.45	0	Fixed	11.72(2.44, 20.99)	0.01
<8 w	4	0.009	74	Random	3.02(−7.62, 13.67)	0.58

PEF% pred						
≥8 w	8	<0.0001	80	Random	4.94(1.07, 8.82)	0.01
<8 w	3	0.77	0	Fixed	3.18(−1.91, 8.28)	0.22

FVC% pred: pulmonary function indices include forced vital capacity to predicted value ratio; FEV1% pred: forced expiratory volume at 1 s to predicted value ratio; FEF25–75% pred: forced expiratory flow between 25% and 75% of vital capacity ratio; PEF% pred: peak expiratory flow to predicted value ratio; SMD: standardized mean difference; MD: mean difference.

## Data Availability

The data used to support the findings of this study are included within the supplementary information file.

## Supplementary Materials

Supplementary Materials Figure S1: risk assessment of bias in RCT. Figure S2: overall risk assessment of bias in RCT. Figure S3: the effect of exercise on 6MWT. Figure S4: the effect of exercise on RPE. Figure S5: the effect of exercise on PP.
