# Supplementary figures and images for: Effects of Physical Exercises on Pulmonary Rehabilitation, Exercise Capacity, and Quality of Life in Children with Asthma: A Meta-Analysis
Source: Evid Based Complement Alternat Med. 2021 Dec 23;2021:5104102. doi: 10.1155/2021/5104102 (PMC8718301; doi:10.1155/2021/5104102)

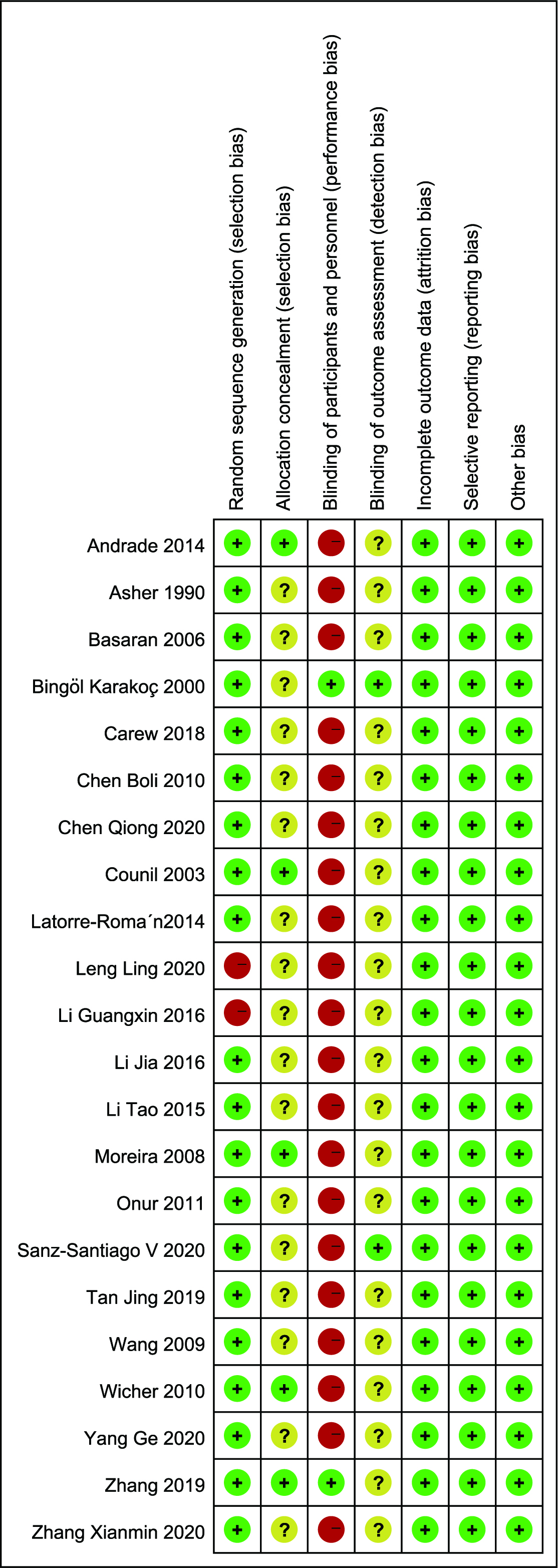

Supplement: Supplementary Materials — Figure S1: risk assessment of bias in RCT. Figure S2: overall risk assessment of bias in RCT. Figure S3: the effect of exercise on 6MWT. Figure S4: the effect of exercise on RPE. Figure S5: the effect of exercise on PP. [file 5104102.f1.zip › 5104102.f1/Fig. S1. Risk assessment of bias in RCT (1).jpg]

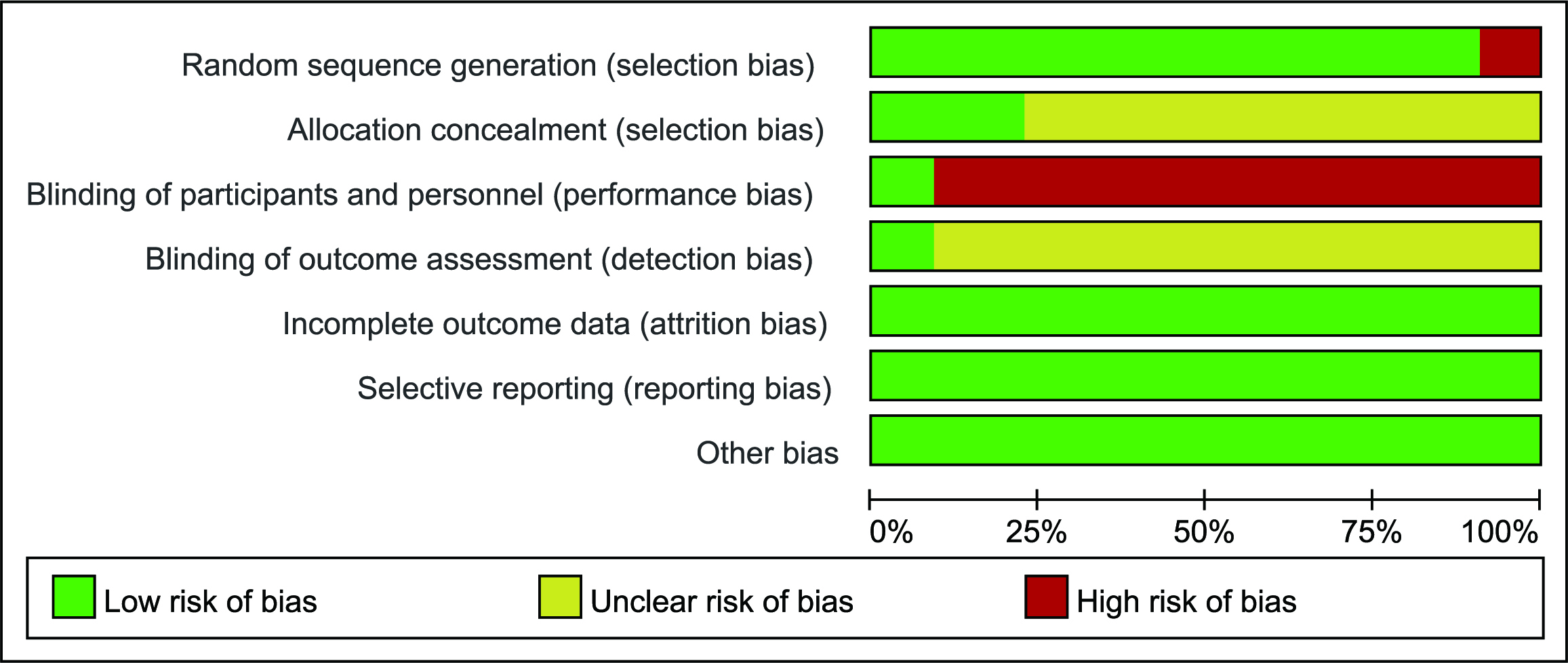

Supplement: Supplementary Materials — Figure S1: risk assessment of bias in RCT. Figure S2: overall risk assessment of bias in RCT. Figure S3: the effect of exercise on 6MWT. Figure S4: the effect of exercise on RPE. Figure S5: the effect of exercise on PP. [file 5104102.f1.zip › 5104102.f1/Fig. S2. Overall risk assessment of bias in RCT (1).jpg]

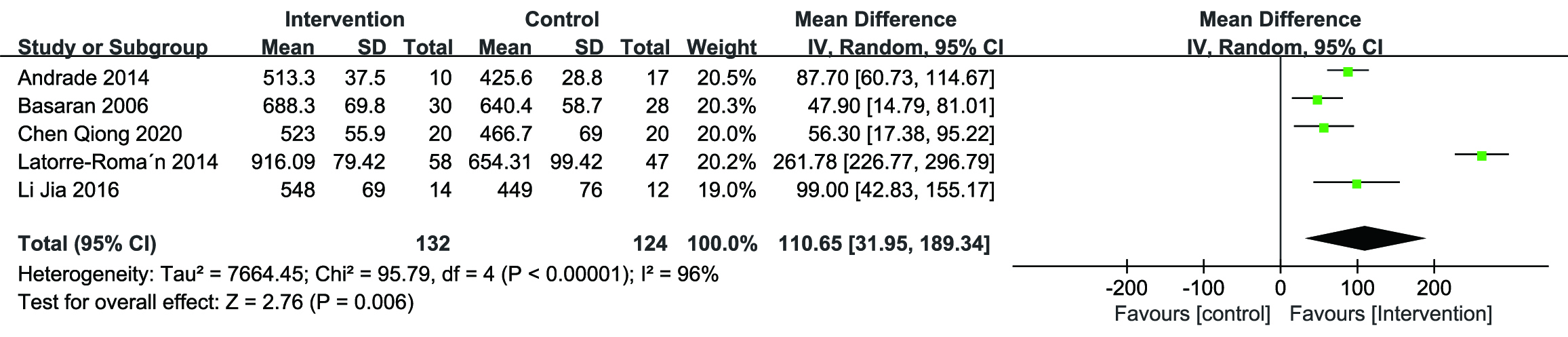

Supplement: Supplementary Materials — Figure S1: risk assessment of bias in RCT. Figure S2: overall risk assessment of bias in RCT. Figure S3: the effect of exercise on 6MWT. Figure S4: the effect of exercise on RPE. Figure S5: the effect of exercise on PP. [file 5104102.f1.zip › 5104102.f1/Fig. S3. The effect of exercise on 6MWT (1).jpg]

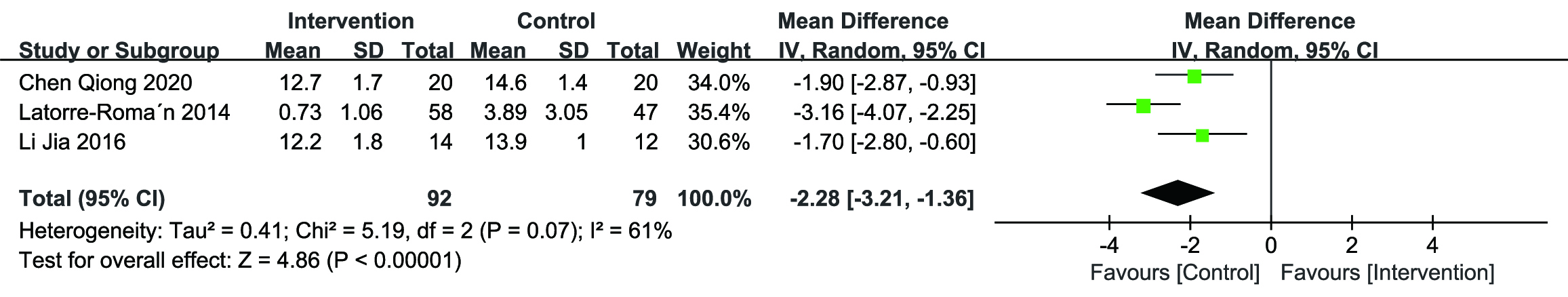

Supplement: Supplementary Materials — Figure S1: risk assessment of bias in RCT. Figure S2: overall risk assessment of bias in RCT. Figure S3: the effect of exercise on 6MWT. Figure S4: the effect of exercise on RPE. Figure S5: the effect of exercise on PP. [file 5104102.f1.zip › 5104102.f1/Fig. S4. The effect of exercise on RPE (1).jpg]

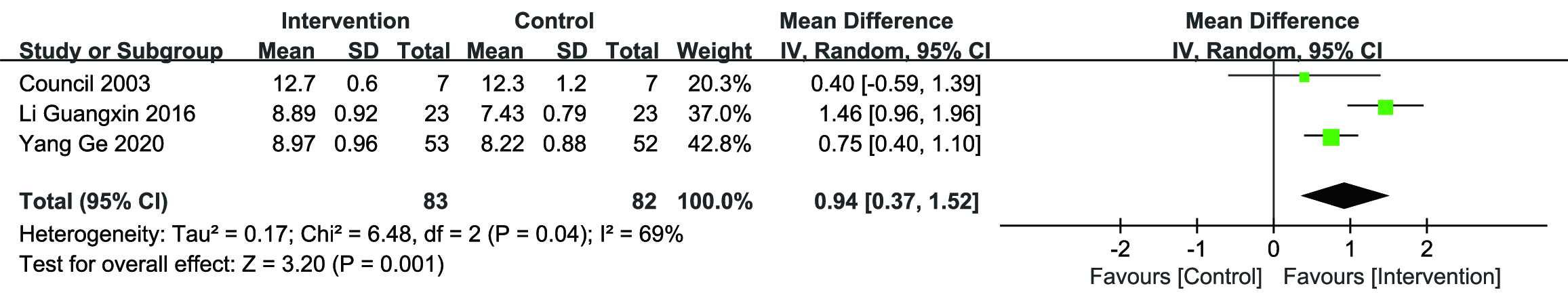

Supplement: Supplementary Materials — Figure S1: risk assessment of bias in RCT. Figure S2: overall risk assessment of bias in RCT. Figure S3: the effect of exercise on 6MWT. Figure S4: the effect of exercise on RPE. Figure S5: the effect of exercise on PP. [file 5104102.f1.zip › 5104102.f1/Fig. S5. The effect of exercise on PP (1).jpg]
